# Cost-Sensitive YOLOv5 for Detecting Surface Defects of Industrial Products

**DOI:** 10.3390/s23052610

**Published:** 2023-02-27

**Authors:** Ben Liu, Feng Gao, Yan Li

**Affiliations:** School of Mechanical and Precision Instrument Engineering, Xi’an University of Technology, Xi’an 710048, China

**Keywords:** defect detection, cost-sensitive learning, YOLOv5, misclassification risk, intelligent industry

## Abstract

Owing to the remarkable development of deep learning algorithms, defect detection techniques based on deep neural networks have been extensively applied in industrial production. Most existing surface defect detection models assign equal costs to the classification errors among different defect categories but do not strictly distinguish them. However, various errors can generate a great discrepancy in decision risk or classification costs and then produce a cost-sensitive issue that is crucial to the manufacturing process. To address this engineering challenge, we propose a novel supervised classification cost-sensitive learning method (SCCS) and apply it to improve YOLOv5 as CS-YOLOv5, where the classification loss function of object detection was reconstructed according to a new cost-sensitive learning criterion explained by a label–cost vector selection method. In this way, the classification risk information from a cost matrix is directly introduced into the detection model and fully exploited in training. As a result, the developed approach can make low-risk classification decisions for defect detection. It is applicable for direct cost-sensitive learning based on a cost matrix to implement detection tasks. Using two datasets of a painting surface and a hot-rolled steel strip surface, our CS-YOLOv5 model outperforms the original version with respect to cost under different positive classes, coefficients, and weight ratios, but also maintains effective detection performance measured by mAP and F1 scores.

## 1. Introduction

Industrial and manufacturing object detection has been greatly facilitated by deep neural networks [1,2,3,4]. Object detection models are exploited to predict the position of the object in the input visual data and the corresponding class information, which plays a role in accelerating intelligent industry transformation [5,6,7]. The YOLO family [8,9] is one of the most popular approaches in real applications due to their fast inference speed and outstanding accuracy. Moreover, in the industrial sector, product surface defect detection is a general task [10,11,12]. To improve productivity, many manufacturing industries utilize machine vision systems, within which object detection is the core algorithm [13].

After all, there is no object detection model that can be completely accurate. The vast majority of existing surface defect detection approaches assume the same cost for all detection errors and focus on realizing high detection accuracy [11,14]. However, previous studies have confirmed that different types of detections and misclassifications lead to distinct costs [15,16,17], which leads to the cost-sensitive problem of models in practical industrial applications. For example, a defect detection model for automobile parts may judge nondestructive parts as defective parts, resulting in the loss of time and efficiency. Conversely, the misclassification of defective parts as nondestructive components may be detrimental to the safety of vehicle users and cause potential risks. Obviously, the cost (decision risk) of the latter is significantly higher than the former. Detection methods based on deep learning have achieved great success [18,19], but most of them cannot directly deal with the cost-sensitive problem, which is an urgent issue needing further investigation. This work mainly focuses on the cost-sensitive defect detection problem caused by discriminative misclassification errors.

Cost-sensitive learning has attracted much attention in past years [17,20,21,22]. Existing cost-sensitive models can be roughly divided into two categories: external cost-sensitive and internal cost-sensitive methods [23,24,25]. The objective of the external cost-sensitive method is to deal with the problem of model discriminant bias related to imbalanced data such as the long-tailed distribution of training datasets [26,27,28,29]. Differently, the internal cost-sensitive method is aimed at the decision cost (risk) caused by the classification errors among different categories in specific application scenarios [24,30]. Recently, these methods have been applied to scenarios such as face recognition, intelligent decisions, and intelligent healthcare [16,31,32,33,34,35,36]. However, most existing deep learning-based object detection work does not consider the cost-sensitive problem, but treats the misclassification cost between different classes equally, which may lead to high decision risk and is not applicable to cost-sensitive surface defect detection.

To address the above internal cost-sensitive issue in defect detection, a supervised cost-sensitive YOLOv5 detection model (CS-YOLOv5) is proposed. Considering the insufficiency of after-processing cost-sensitive methods, a direct-type cost-sensitive principle is conducted after re-examining the training process of object detection. Such a principle requires the output class prediction results of the defect detection model to be directly cost-sensitive, rather than being computed in an additional decision stage. That is, the parameters of the object detection model should preserve the classification cost information. The main contributions are listed as follows:

- Therefore, a classification loss function based on a label–cost vector selection method is designed, which can equip YOLOv5 with cost sensitivity after training. The misclassification cost involved might be the labor cost of defect detection, security cost, etc., which can be specified in practice by defining the cost matrix.
- Compared with the original YOLOv5 model, CS-YOLOv5 can solve the internal cost-sensitive problem, exploiting the classification risks defined by a risk matrix in specific applications.
- Experiments on our newly constructed painting defect dataset as well as a hot-rolled steel strip defect dataset demonstrate the superiority of our approach.

The rest of the paper is organized as follows. In Section 2, a newly developed principle and model are proposed. In Section 3, experiments and results analysis are reported to verify the effectiveness of the new model while discussing the proposed method. Finally, Section 4 concludes this paper.

## 2. Methodology

**Figure 1:**
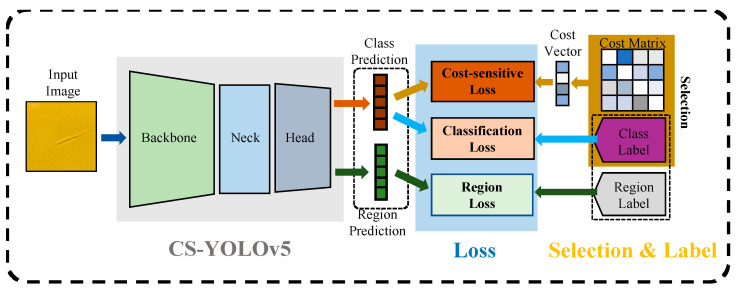
CS-YOLOv5 model and classification loss structure.

Figure 1 illustrates the CS-YOLOv5 model and its classification loss structure proposed in this paper. To exploit defect misclassification risks described by a cost matrix, a direct-type cost-sensitive principle based on a label–cost vector selection was designed. Under this principle, we propose a new cost-sensitive loss function. Specifically, the input defect images in training go through the forward process of the deep neural network and are encoded to class predictions and region predictions. Then, cost vectors are selected from the cost matrix via supervised class labels, which are utilized for the cost-sensitive loss calculation along with the class prediction. Meanwhile, classification loss and region loss are also derived. Finally, an object detection model with cost-sensitive features is obtained by backpropagating the loss gradient and updating the model parameters.

### 2.1. Cost-Sensitive Learning Modeling

Internal cost-sensitive learning attempts to reduce the decision risk defined in specific scenarios in training. This can be formulated as follows [37]:

(1) $$\phi^{*}\left(x\right)=\arg\underset{j}{min}\mathrm{loss}\left(x,j\right)$$

where loss(x,j)  is the expected cost of classifying sample x as *j*-th class, which is determined by the element *C_ij_* in the cost matrix ***C***. Furthermore, the cost matrix ***C*** is obtained via analysis from domain experts or data mining, e.g., estimating the cost of leakage and security costs in surface defect detection.

Without loss of generality, in deep learning, the classification loss is expressed as a function of the difference between the supervised class labels and the output prediction distribution. Assuming X and D are the input space and the output space, respectively, then the expectation of classification error can be expressed as:

(2) $$E=E_{X,D}l\left[\varphi \left(X\right),D\right]$$

where φ(X) is the prediction result of the model for input space X, and l(⋅) is the measurement method of the classification error. Therefore, the classification error for a single input sample x is described as follows:

(3) $$e_{x,d}=l\left(p,d\right)$$

where $p=\varphi \left(x\right)=\left[p_{1},\cdots ,p_{m}\right]$ is the category probability vector estimated by the model for x, and the element *p_j_* denotes the probability of the input sample x being recognized as the *j*-th class. Similarly, ***d*** is the one-hot label vector of x. For a sampled batch *b* with size *B*, Formula (2) can be expressed as:

(4) $$E_{b}=\frac{1}{B}\sum_{i}^{B}l\left(p^{\left(i\right)},d^{\left(i\right)}\right)$$

In terms of a defect detection model, p=g(x,θ), where g(⋅,θ) is the deep neural network with a parameter set of *θ*. The general classification loss function can measure the difference between the input space and the output space. However, there is no specific design for the output space. Classification errors between different classes are treated equally, without cost sensitivity. Generally, the total loss function includes the classification loss and a loss related to the region predictions for targets. Therefore, the general form can be expressed as follows:

(5) $$L=L_{cls}+\beta L_{reg}$$

where *L_cls_* is the classification loss designed in a cross-entropy form, *L_reg_* is the location region loss, and *β* is a trade-off parameter.

According to the calculation of the ordinary classification loss described by Equation (4), the following conclusions can be drawn: (1) The classification loss between different categories is not assigned specifically. (2) Moreover, only the difference between class predictions and the correspondence labels is taken into account. In essence, it is still a generic non-cost-sensitive loss function that cannot process misclassification risks in defect detection.

The classification cost matrix ***C*** (decision risk matrix) is formulated as:

(6) $$\left[\begin{array}{llll} 0 & C_{12} & \cdots & C_{1N}\\ C_{21} & 0 & \cdots & C_{2N}\\ \vdots & C_{i2} & 0 & \vdots \\ C_{N1} & \cdots & C_{N\left(N-1\right)} & 0 \end{array}\right]$$

The number of rows and columns of ***C*** are equal to the classes’ quantity *N*, where $C_{ij}⩾0$ and $C_{i}\neq 0$. Without loss of generality, $C_{ii}=0$ means that the classification is correct without cost. Row vector ***C_i_*** denotes the cost of classifying the samples with the class i into each class [30,38]. In multiclass detection or classification tasks, if all classes are divided into positive classes and negative ones, the misclassification coefficient can be divided into 4 types [16]:

1. False acceptance coefficient *λ_NP_*: the risk of misclassifying a target (imposter) that belongs to the negative category into the positive category or positive class (gallery).
2. False rejection coefficient *λ_PN_*: the risk of misclassifying a gallery to an imposter.
3. Two types of misidentification coefficients *λ_PP_* and *λ_NN_*: that is, misclassification between classes of samples with the same nature (the positive class or negative class).

The relationship between the magnitudes of the different coefficients is generally expressed as follows:

(7) $$\lambda_{NP}>\lambda_{PN}>\lambda_{NN}>\lambda_{PP}$$

Moreover, the elements of the cost matrix can be expressed as:

(8) $$C_{ij}=\{\begin{array}{lll} \lambda_{NP}, & \;\mathrm{if}\; & i\epsilon N,j\epsilon P\\ \lambda_{PN}, & \;\mathrm{if}\; & i\epsilon P,j\epsilon N\\ \lambda_{NN}, & \;\mathrm{if}\; & i\epsilon N,j\epsilon N\\ \lambda_{PP}, & \;\mathrm{others}\; & \end{array}$$

### 2.2. Direct-Type Cost-Sensitive Learning

The goal of cost-sensitive classification is to learn the probability prediction vectors formulated in Equation (4), which not only has high accuracy defined by supervised labels but also low cost with respect to the cost matrix ***C***. In this work, we argue that the cost matrix is additional supervised information for object detection. The rationale behind this is that the cost matrix reflects requirements for the model in a specific scenario. Therefore, we describe how to train a cost-sensitive defect detection model using both kinds of supervised information in the following part.

As the label exploitation is already formulated in Equation (4), we construct the cost-sensitive constraint in a label-cost vector selection method. Specifically, the cost vector is selected from the cost matrix ***C*** via the label ***d*** of the input sample x. This vector also constrains the output of posterior probability ***p***, in which the maximum probability in ***p*** determines the output class. Obviously, our approach incorporates cost information directly into training, which is different from the post-processing cost-sensitive learning based on Bayes minimum risk methods [31,39].

In the case of supervised learning, the ground-truth class index of sample x with label ***d*** is:

(9) $$t=\underset{1⩽i⩽N}{\arg max}d_{i}$$

According to Equation (1), the target of direct-type cost-sensitive learning is formulated as:

(10) $$\{\begin{array}{c} p^{\prime }=\underset{p^{\prime }\in {\mathbb{R}}^{N}}{\arg min}p^{\prime }\cdot C_{t}\\ \phi^{*}\left(x\right)=\underset{1⩽i⩽N}{\arg max}p^{\prime } \end{array}$$

where $p^{\prime }$ is the output probability vector, and ***C****_t_* is the aforementioned selected risk (cost) vector. Let *R_tp_*(*x*) represent the misclassification risk of samples of class *t* under a current probability vector ***p***. The risk brought by ***p*** can be expressed as:

(11) $$\{\begin{array}{l} p^{\prime }=\underset{p^{\prime }\in {\mathbb{R}}^{N}}{\arg min}R_{tp^{\prime }}\left(x\right)=\underset{p^{\prime }\in {\mathbb{R}}^{N}}{\arg min}\sum_{j=1}^{N}p_{j}^{\prime }C_{tj}\\ \phi^{*}\left(x\right)=\underset{1⩽i⩽N}{\arg max}\left(p_{i}^{\prime }\right) \end{array}$$

Equation (11) indicates that in the training of the direct-type cost-sensitive model: (1) output vector $p^{\prime }$ is to minimize the overall misclassification cost, i.e., minimizing the risk of classifying the sample belonging to class *t* into each class. (2) The same as before, the maximum index in the probability vector determines the class output. This is the direct-type cost-sensitive learning principle of the deep learning model with supervised label and cost information. The new principle specifies the calculation form of risk under supervised learning and provides linear constraints for the estimation of probability directly in N-dimensional space.

### 2.3. CS-YOLOv5 Model and Method Analysis

In order to equip the defect object detection model with cost sensitivity, the modified loss function is proposed and described as follows:

(12) $$\begin{array}{cc} \mathrm{Loss} & =\lambda_{iou}\sum_{i=0}^{S^{2}}\sum_{j=0}^{B}1_{ij}^{obj}L_{\mathrm{CIoU}}\\ \; & -\lambda_{cls}\sum_{i=0}^{S^{2}}\sum_{j=0}^{B}1_{ij}^{obj}\sum_{c\epsilon classes}\lambda_{c}\left[{\hat{p}}_{ti}\left(c\right)\log\left(p_{i}\left(c\right)\right)+\left(1-{\hat{p}}_{ti}\left(c\right)\right)\log\left(1-p_{i}\left(c\right)\right)\right]\\ \; & +\eta \lambda_{\mathrm{cost}\;}\sum_{i=0}^{S^{2}}\sum_{j=0}^{B}1_{ij}^{obj}\sum_{c\epsilon \;\mathrm{llasses}\;}\lambda_{c}S\left(p_{i}\left(c\right)\right)C_{[t(ij)]c}\\ \; & +\lambda_{obj}\sum_{i=0}^{S^{2}}\sum_{j=0}^{B}1_{ij}^{obj}\lambda_{p}\left[{\hat{C}}_{i}\log\left(C_{i}\right)+\left(1-{\hat{C}}_{i}\right)\log\left(1-C_{i}\right)\right]\\ \; & +\lambda_{obj}\sum_{i=0}^{S^{2}}\sum_{j=0}^{B}1_{ij}^{\mathrm{noobj}\;}\left[{\hat{C}}_{i}\log\left(C_{i}\right)+\left(1-{\hat{C}}_{i}\right)\log\left(1-C_{i}\right)\right] \end{array}$$

where $C_{\left[t\left(ij\right)\right]c}$ is the cost of that the *ij*-th sample of the class t(ij) is classified into ***C*** class, and is the *c*-th element of cost vector $C_{\left[t\left(ij\right)\right]}$ from the risk coefficient matrix ***C***. *η*, $\lambda_{\mathrm{cost}\;}$, $\lambda_{cls}$, $\lambda_{iou}$, and $\lambda_{obj}$ are trade-off parameters that control the importance of distinct parts of losses. $1_{ij}^{obj}$ returns the value of 1 if the *ij*-th region contains an object. $S\left(y\right)={\left(1+e^{-y}\right)}^{-1}$ is the sigmoid logistic regression function. Individually, the new classification loss function is expressed as:

(13) $$\begin{array}{ll} L_{cls} & =\eta \lambda_{\mathrm{cost}}\sum_{i=0}^{S^{2}}\sum_{j=0}^{B}1_{ij}^{obj}\lambda_{c}⊙S\left(p_{i}\right)\cdot C_{\left[t\left(ij\right)\right]}\\ & -\lambda_{cls}\sum_{i=0}^{S^{2}}\sum_{j=0}^{B}1_{ij}^{obj}\sum_{c\epsilon \;\mathrm{lasses}\;}\lambda_{c}\left[{\hat{p}}_{ti}\left(c\right)\log\left(p_{i}\left(c\right)\right)+\left(1-{\hat{p}}_{ti}\left(c\right)\right)\log\left(1-p_{i}\left(c\right)\right)\right] \end{array}$$

where the first term is the cost-sensitive classification loss. When the parameter $\lambda_{\mathrm{cost}}$ = 0 and $\lambda_{cls}\neq 0$, the loss degenerates to a non-cost-sensitive loss. When $\lambda_{\mathrm{cost}}\neq 0$ and $\lambda_{cls}=0$, the loss degradation calculation is similar to the loss function in [25]. However, the intuitive meaning of improving the loss function in [25] is that a $O_{2}$ item is sufficiently close to the label value, which does not follow the cost-sensitive principle in Equation (10). The cost-sensitive principle in Equation (10) is further formulated as:

(14) $$L_{\mathrm{cost}}=\sum_{\mathrm{ccclasses}\;}\lambda_{c}S\left(p_{i}\left(c\right)\right)C_{tc}\;$$

The use of a sigmoid function and normalization operation for the risk matrix in practical calculation can guarantee the boundary, thereby preventing the vanishing gradient and gradient explosion in training. In Equation (13), the classification loss is constructed as minimizing classification risk under class labels and the cost matrix. In other words, the optimization of the cross-entropy loss ensures the accuracy of the output in the label sense, that is, the estimate of the posterior probability is close enough to the label. The existence of cost-sensitive classification loss ensures that the risk corresponding to the estimated posterior probability is small enough. The loss weights of both realize the balancing effect of classification loss for different parts.

#### 2.3.1. Cost-Sensitive Gradients

During the supervised training of the object detection model, the impact of the modified classification loss function on the implementation of the backpropagation algorithm needs to be taken into consideration [40]. Since the cross-entropy loss in the classification loss function remains unchanged and is superimposed with the cost-sensitive loss function, it will not affect the original backpropagation process. For a sample x of class *t*, the estimate of its output probability is set as ***p***. For the output layer, the gradient with respect to output neuron 0*_n_* is directly associated with the *n*-th dimension output *p_n_* and can be expressed as:

(15) $$\frac{\partial L_{\mathrm{cost}\;}}{\partial o_{n}}=\lambda_{cn}C_{tn}\frac{\partial S\left(p_{n}\right)}{\partial p_{n}}$$

Since $\frac{\partial S\left(y\right)}{\partial y}=S\left(y\right)\left(1-S\left(y\right)\right)=\frac{e^{-y}}{{\left(1+e^{-y}\right)}^{2}}$, then:

$$\frac{\partial L_{cost}}{\partial o_{n}}=\lambda_{cn}C_{tn}\frac{e^{-p_{n}}}{{\left(1+e^{-p_{n}}\right)}^{2}}$$

The gradient of the cost-sensitive loss function directly with respect to the output probability is:

(16) $$\nabla L_{cost}=\sum_{n=1}^{N}\lambda_{cn}C_{tn}\frac{e^{-p_{n}}}{{\left(1+e^{-p_{n}}\right)}^{2}}$$

Since $e^{-p_{n}}>0$ and $C_{i}\neq 0$, then $\nabla L_{\cos t\;}\neq 0$.

#### 2.3.2. Cost-Sensitive YOLOv5 Algorithm

The role of the cost-sensitive classification loss function proposed in this paper is to establish a cost-sensitive parameter optimization space by combining various loss functions. The optimization algorithm is executed on this parameter space, thereby continuously updating the weight parameters of the model. The forward propagation of the established CS-YOLOv5 model has cost sensitivity. The implementation process is expressed in Algorithm 1:
**Algorithm 1: Training direct-type cost-sensitive YOLOv5****Input**: training space (X, D), validation set $\left(X_{v},D_{v}\right)$, minibatch size b, training epochs e, learning rate *lr*, as well as loss weight $\left(\lambda_{cost},\lambda_{cls},\lambda_{iou},\lambda_{c}\right)$, and model *g*(*θ*) **Output:** trained model *g*(θ*) 1: Initialize parameters randomly of the model *g*(*θ*_0_) 2: If the current epoch i⩽e, loop: 3:   If the current batch is *j*, loop: 4:    Forward propagation: $O_{j}=g\left(X_{j},D_{j}|\theta_{j-1}\right)$ 5:    Calculate the loss $L_{j}=\left(O_{j},D_{j}\right)$ based on Equation (12) 6:    Calculate the gradient $\nabla L_{j}$ and backpropagation, execute the SGD algorithm 7:    Update parameter $\theta_{j}\leftarrow \theta_{j-1}$ 8:   Evaluate the model: $g\left(X_{v},D_{v}|\theta_{i}\right)$ 9: Return $g\left(\theta^{*}\right)$
**End**

Through the proposed cost-sensitive loss method, the misclassification cost information is directly involved in the training of the deep neural network model. With the classification loss weight $\left(\lambda_{\mathrm{cost}},\lambda_{cls}\right)$ set within an appropriate range, we have the following observations: 

(Case 1): When the risk coefficient of the cost vector is *C_ii_* = 0 or *C_ii_* is the smallest element in vector ***C_i_***, classifying the sample into its ground-truth has the lowest risk. If the risk coefficient *C_in_* is corresponding to the large value *p_n_* in the output probability, the classification cost will be large. The model optimization process results in a delineation of the ground-truth and drives away this costly situation.

(Case 2): When the risk coefficient *C_ii_* of the cost vector is the element with a larger value in the vector ***C_i_*** and the risk coefficient corresponding to the larger value *p_n_* in the posterior probability estimation is exactly *C_ii_*, the correct classification will cause a large classification cost. After parameter updating, the model optimization process will lead to the division of clear truth value label pointing.

The essence of the classification loss function is to move away or strengthen the decision boundary towards the direction of less risk. Then, the boundary ambiguity is reduced compared with non-cost-sensitive models. From another perspective, the method proposed in this paper uses the prior knowledge of the cost matrix to modify the constraints of the model optimization. Thus, the optimization of model parameters can be regarded as a regularization method. The cost-sensitive learning with the original Bayes minimum risk decision type is to simply concatenate the output of estimating probability through the process of the minimum-risk decision. Instead, our new cost-sensitive learning method proposed in this paper is to obtain the optimal posterior probability vector in the sense of the label and cost matrix in one training process, which makes the model output directly cost-sensitive.

## 3. Experiments and Results Analysis

### 3.1. Experimental Dataset

NEU surface defect database: This industrial image dataset of hot-rolled steel strip surface defects is constructed in [41], which contains six types of defects, i.e., rolled-in scale (RS), patches (Pa), crazing (Cr), pitted surface (PS), inclusion (In), and scratches (Sc). There are 300 images in each class for a total of 1800 images. In the experiment, 1500 images were divided into a training set, and the remaining 300 images were divided into a test set. The original data size was 200 × 200 pixels, which was converted to size 192 × 192 in the experiments. This dataset is referred to as the NEU dataset for simplicity in this paper.

Paint surface defect dataset: This is a manufacturing image dataset involving paint spraying surface defects. The author participated in the collection and collation work of the inspection site of construction machinery structural parts. The dataset contains four classes of target defects, including dust, pit, sag, and scratch. There were 1417 images in total, including 1246 training set images and 171 test set images. The original size was 1024 × 1024, which was set to size 640 × 640 in the experiments. For simplicity, this dataset is referred to as the Paint dataset in the remainder of this paper.

**Figure 2:**
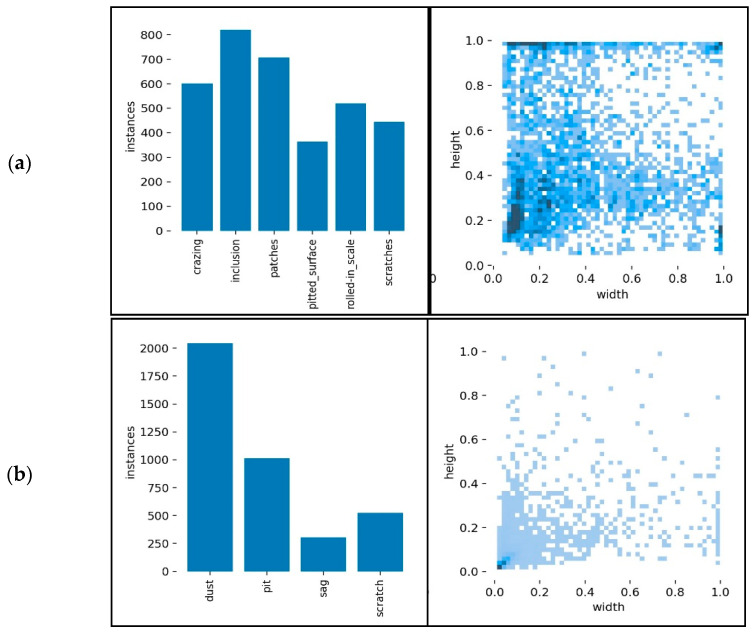
(**a**) NEU dataset sample statistics (**b**) Paint dataset sample statistics.

The class distributions and the size distribution of ground-truth bounding box of these two datasets are counted and shown in Figure 2a,b, respectively. In the bar figures, the values denote the statistics of the sample numbers for each class. For the NEU dataset, the number of sample classes is more uniform than Paint. The Paint dataset has obvious uneven sample distribution, in which sag and scratch are less than the other two classes, and most of the two classes are more than 75% of the total number of samples. This is because the frequency of sag and scratch is so low at the collection site. Thus, it is a long-tail problem, which is very common in application.

The scatter figures exhibit the relative size distribution of ground-truth bounding box for the datasets, that is, the distribution of length and width. The darker color indicates more bounding boxes are concentrated here. It can be seen that the bounding box width and height of the NEU dataset are widely distributed. The densest is where the relative width is about 0.2 and the relative height is about 0.1. In the Paint dataset, the bounding box is clearly concentrated in the lower left corner of the scatterplot. This is because the actual bounding boxes of dust samples in the dataset are relatively small, and the corresponding number is much larger than other classes. Based on the above analysis, the Paint dataset has obvious sample imbalance and most sample bounding boxes are too small. Therefore, the object detection problem of the Paint dataset is more difficult than that of the NEU dataset.

### 3.2. YOLOv5 Model Settings

**Figure 3:**
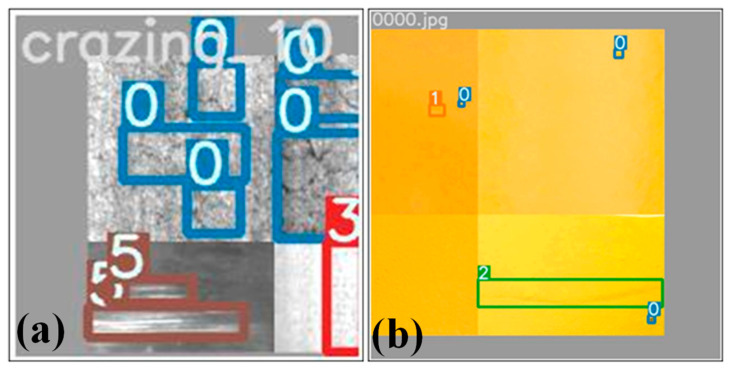
(**a**) NEU Data augmentation (**b**) Paint Data augmentation. Numbers are class indexes.

YOLOv5 was selected as the baseline model in experiments, whose code is provided by [7]. We experimented with several Tesla T4 GPUs and implemented the code with the Pytorch 1.7.1 platform. The initial learning rate was set to 0.002. The cosine annealing strategy was utilized during training. The weight decay coefficient was 0.0005 for the adopted SGD optimizer. The balance parameter *β* was set to 0.375, and *η* was set to 1/10,000. For the NEU dataset, the batch size was set to 230, with 90 epochs of training. For the Paint dataset, the batch size was set to 24, with 55 epochs of training. The experiments all used the Mosaic data augmentation method. As the examples show in Figure 3a,b, this augmentation method stitches images together in an overlapping manner to improve the robustness of the models.

### 3.3. Experimental Metrics 

#### 3.3.1. Classification Cost Evaluation Metric

The proposed cost-sensitive learning method concentrates on minimizing the decision risk within the acceptable range of accuracy. To quantitatively measure the classification cost, we construct a metric formulated as:

(17) $$\mathrm{Cost}\;=\frac{1}{F}\sum_{i=1}^{F}\sum_{c\in \;\mathrm{cesses}\;}p_{ic}C_{\left[t\left(i\right)\right]c}=\frac{1}{F}\sum_{i=1}^{F}P_{i}\cdot C_{\left[t\left(i\right)\right]}$$

where $C_{\left[t\left(i\right)\right]c}$ is the *c*-th element of the cost vector $C_{\left[t\left(i\right)\right]}$ from matrix ***C*** and denotes the cost of classifying the *j*-th sample belonging to the class t(i) into the c-th class. *F* is the number of positive samples used to calculate the loss. Intuitively, such a cost metric is consistent with the cost-sensitive target to be optimized in the loss function.

#### 3.3.2. Comprehensive Metrics

The recall ratio in object detection refers to the proportion of data samples correctly detected by the model, which is one of the basic performance measurements. TP, FP, TN, and FN are true positive, false positive, true negative, and false negative examples, respectively. Then, the recall rate is:

(18) $$\mathrm{Recall}\;=\frac{\mathrm{TP}}{\mathrm{TP}+\mathrm{FN}}$$

To comprehensively evaluate the trained models, we also adopted the common metric, mean average precision (mAP) and F1-score in the experiments. They are described as follows:

(19) $$\mathrm{mAP}=\int_{0}^{1}\mathrm{Precision}\left(r\right)dr$$

where Precison =TP/(TP+FP) is the accuracy rate, and *r* represents Recall.

(20) $$F1=2\times \frac{\;\mathrm{Precision}\;\cdot \;\mathrm{Recall}\;}{\;\mathrm{Precision}\;+\;\mathrm{Recall}\;}$$

For the multiclass data (more than two), the calculation method is given in the literature [42]. 

### 3.4. Risk Coefficient Experiment

The premise of cost-sensitive learning methods is to construct a classification cost matrix for the current data. Since the cost matrix is directly involved in the calculation of the classification cost of Equation (16), we performed experiments with different classification cost matrices. Different risk coefficient ratios simulate different setting conditions of the cost matrix in real applications to verify the effectiveness of the cost-sensitive learning method.

Each element of the cost matrix is the classification risk coefficient. Moreover, objects in the dataset were divided into a positive class and a negative class. The settings of the four risk coefficients follow Equation (7). In this experiment, we tested the cost-sensitive learning method by adjusting the rate of risk coefficient under fixed positive classes.

#### 3.4.1. Risk Coefficient Setting

**Figure 4:**
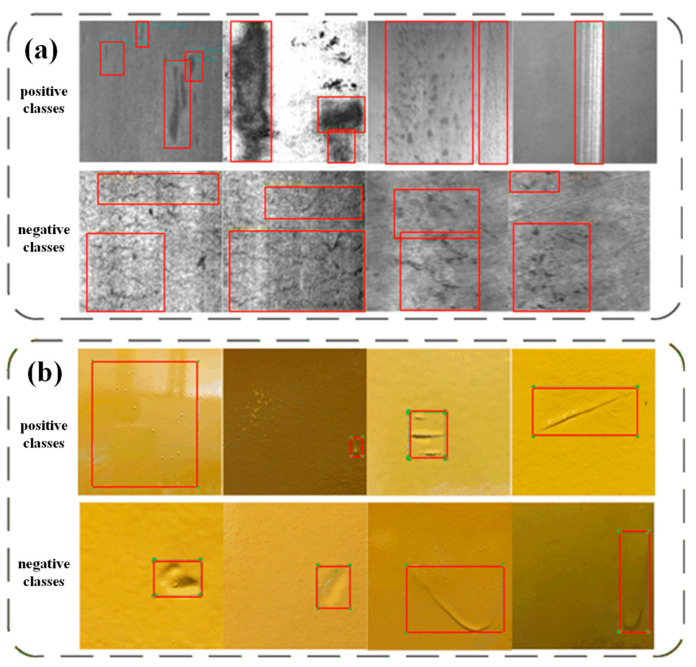
(**a**) Example of NEU division (**b**) Example of Paint division.

For the NEU dataset, positive classes were selected as Pa, PS, In, and Sc, while for Paint, dust and scratch were positive classes. Figure 4a,b shows the examples of positive and negative classes in NEU and Paint dataset, respectively.

According to Equation (7), the base coefficient is selected as $\lambda_{NP}:\lambda_{PN}:\lambda_{NN}:\lambda_{PP}=200:20:2:1$. The ratios between adjacent coefficients are linearly scaled by equal proportions to obtain four sets of classification risk coefficients. Table 1 shows the risk coefficient grouping.

#### 3.4.2. Experimental Results and Analysis

**Figure 5:**
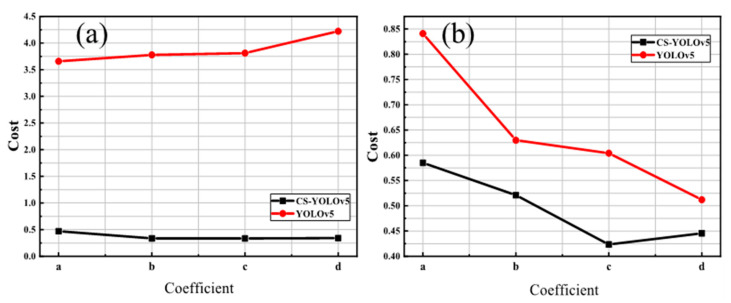
(**a**) Cost (×10^−3^) with varying risk coefficients on NEU (**b**) Cost (×10^−3^) with varying risk coefficients on Paint.

The experimental results of classification cost of four groups (a)–(d) with different risk coefficients are shown in Figure 5. For the four groups (a), (b), (c), and (d), the classification costs related to CS-YOLOv5 are 0.000470, 0.000337, 0.000340, and 0.000341, while those for YOLOv5 are 0.00368, 0.00378, 0.00381, and 0.00422. Compared with YOLOv5, the cost of group (a) for CS-YOLOv5 decreases by 87.16%, and the most significant decrease in group (d) is 91.93%. This is because the risk coefficient ratio of different groups directly affects the cost matrix, thus giving CS-YOLOv5 different cost sensitivities during training. According to the cost metric given by Equation (17), different risk matrices also make models have different cost performances. Similar conclusions can be obtained in Figure 5b. According to the results of the NEU and Paint datasets, the classification cost of the cost-sensitive YOLOv5 model is also significantly lower than that of the original YOLOv5. Under different risk coefficients, the output of the cost-sensitive YOLOv5 model itself has lower classification costs, which proves the effectiveness of our method.

Moreover, the results of mAP and F1-score are shown in Table 2. The improved model has cost-sensitive capabilities while performing comparably to the original model under two metrics. Even the performance of the proposed approach is better than the original YOLOv5 under some risk coefficients (bold in the table). It can be concluded that when other cost matrix settings such as positive and negative classes are fixed, cost-sensitive YOLOv5 significantly improves the cost-sensitive performance without losing the comprehensive performance.

### 3.5. Experiment of Positive Classes

In real scenarios, positive and negative classes are often determined in accordance with different production requirements. The division of the positive class and negative class plays a crucial role in cost-sensitive learning. According to Section 3.2, the division is directly related to the form of the cost matrix. Therefore, we performed this experiment by setting the number of positive classes as a variable to verify the effectiveness of CS-YOLOv5.

#### 3.5.1. Number Setting of Positive Classes

In this part, the risk coefficient was set to a fixed $\lambda_{NP}:\lambda_{PN}:\lambda_{NN}:\lambda_{PP}=200:20:2:1$. For the NEU dataset, the number of positive classes was increased from 1 to 4. For the Paint (with a total of 4 classes), the number of positive classes was increased from 1 to 3. Considering the preciseness, the positive classes were randomly selected and tested several times.

#### 3.5.2. Experimental Results and Analysis

**Figure 6:**
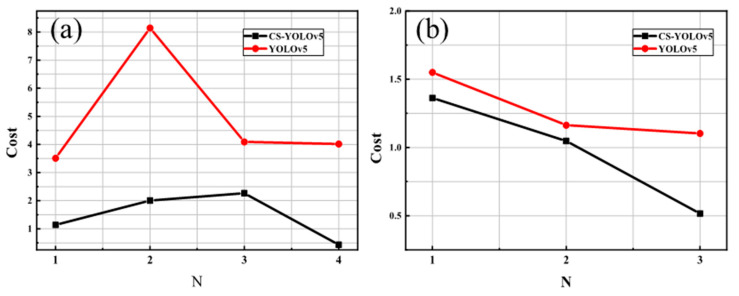
(**a**) Cost (×10^−3^) with varying positive classes on NEU (**b**) Cost (×10^−3^) with varying positive classes on Paint.

For the division, samples belonging to positive classes were classified into the positive set, while samples of other classes are classified into the negative set. The cost matrix conducted based on this division and risk coefficients was exploited in training. The classification cost results under different positive classes are shown in Figure 6.

As can be seen from Figure 6, when the number of positive classes in the NEU surface defect database dataset is 1, 2, 3, and 4, the classification cost of CS-YOLOv5 is 67.58%, 75.36%, 44.56%, and 89.27% lower than that of YOLOv5. Different numbers of positive classes will affect the construction of the cost matrix, so the parameters of CS-YOLOv5 have different cost sensitivity. According to the cost metric defined by Formula (17), the change in the cost matrix will lead to different classification cost of YOLOv5. Similarly, the analysis of Figure 6b can also reach a similar conclusion. Therefore, the classification cost of the CS-YOLOv5 model is lower than the original model under various partitions on both datasets, which demonstrates the superiority of the direct-type cost-sensitive method. In addition, the performance under mAP and F1-score is shown in Table 3.

From these results, we have some observations: (1) CS-YOLOv5 has performance which is comparable to or better than the original model under the metrics. (2) When other settings such as the misclassification risk coefficient are fixed, the cost sensitivity of CS-YOLOv5 is significantly improved.

### 3.6. Weight Ratio Experiment

As stated in Section 3.4, the cost-sensitive classification loss (Equation (14)) conforms to the direct-type cost-sensitive principle proposed for supervised learning. Furthermore, the optimization of the cross-entropy loss in the classification loss function enforces the estimation of the output probability close to the corresponding ground-truth. On the other hand, the role of the cost loss function (Equation (16)) is the total classification risk of the output probability. The proportion and balance of the two targets are determined by the weight ratio $r=\lambda_{\cos t\;}/\lambda_{cls}$. Therefore, it is reasonable and necessary to implement experiments on this weight ratio in the classification loss.

#### 3.6.1. Settings for Weight Ratio Experiment

The risk coefficient is set to $\lambda_{NP}:\lambda_{PN}:\lambda_{NN}:\lambda_{PP}=200:20:2:1$. For the NEU dataset, positive classes are selected as Pa, PS, In, and Sc, and those are dust and scratch for Paint. Moreover, the weight ratio $r=\lambda_{\cos t\;}/\lambda_{cls}$ is set to vary from 0 to 1.5 with an increment of 0.25. A total of 7 groups of linearly changing ratios are considered. According to the analysis in Section 2.3, when r=0, the classification loss used in the experiment degenerates into a non-cost-sensitive loss function. In this case, the model is non-cost-sensitive.

#### 3.6.2. Experimental Results and Analysis

**Figure 7:**
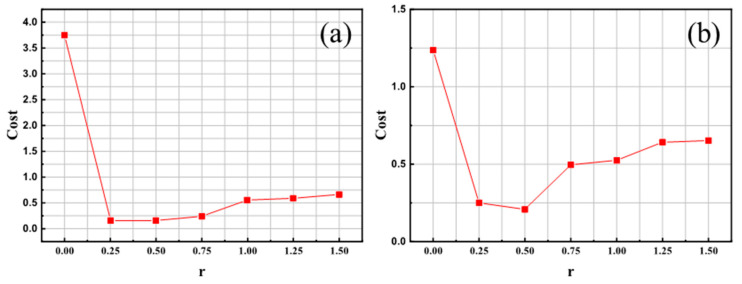
(**a**) Cost (×10^−3^) with varying weight ratios on NEU (**b**) Cost (×10^−3^) with varying weight ratios on Paint.

The experimental results under different loss weight ratios are developed into coefficient weight–classification cost curves in Figure 7. When r=0, the results denote the original non-cost-sensitive model and the other 6 groups of CS-YOLOv5.

As shown in Figure 7a, *r* varies from 0.25 to 1.5 on NEU, representing 6 groups of CS-YOLOv5 models with different loss weight ratios. *r* = 0 indicates that the model degenerates to the original model YOLOv5. From these results, we can see that the classification cost of 6 groups of CS-YOLOv5 models is lower than that of YOLOv5, which indicates the effectiveness of the cost-sensitive method for model improvement. Too-large r can negatively affect the performance of CS-YOLOv5. Thus, in constructing CS-YOLOv5, an appropriate loss weight ratio can equip the model with cost sensitivity and maintain good performance. Similar conclusions can be drawn from the experiments on the Paint dataset in Figure 7b. The cost of the CS-YOLOv5 model on both datasets is lower than the results of the ordinary YOLOv5 model represented by *r* = 0. Consequently, under the cost-sensitive classification functions of different weights in experiments, the effectiveness of the direct-type cost-sensitive method in reducing the classification cost has been demonstrated. In addition, the comprehensive performance measured by mAP and F1-score is shown in Table 4.

From the results on the Paint dataset in Table 4, it can be observed that the improved model with appropriate weight ratio performs better than the original model. When the weight ratios are 0.75 and 0.64, the mAP and F1 metrics reach their optimal values, respectively. As the weight of the cross-entropy loss function decreases, the performance of the model degrades. When the weight ratio is set to 1.5, both mAP and F1 drop to the lowest value of 0.59. This is because a small proportion of cross-entropy loss makes the model parameter more inclined to be cost-sensitive, with insufficient accuracy optimization. Similar conclusions can be drawn from the results of the NEU dataset. Therefore, it can be concluded that the two weights $\lambda_{cost}$ and $\lambda_{cls}$ in the classification loss need to be properly set, so that YOLOv5 can have cost sensitivity and comprehensive performance.

### 3.7. Ablations

As clarified in Section 3.1, the resolution of these two datasets is quite different, which is 192 × 192 for NEU and 640 × 640 for Paint. Thus, the batch sizes on them have a gap under same the computing resources. On another, we implemented a smaller batch size on NEU and found that they had few differences, and the results are shown in Table 5.

**Figure 8:**
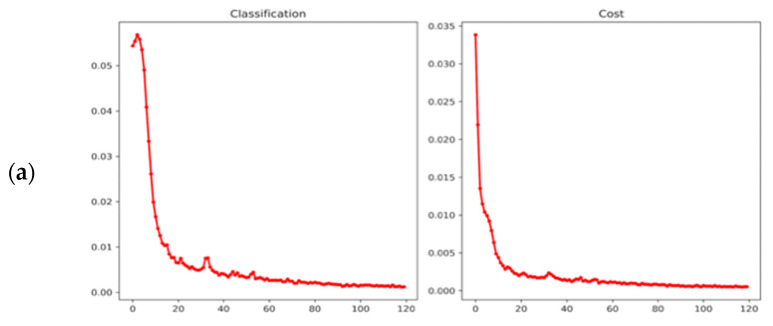

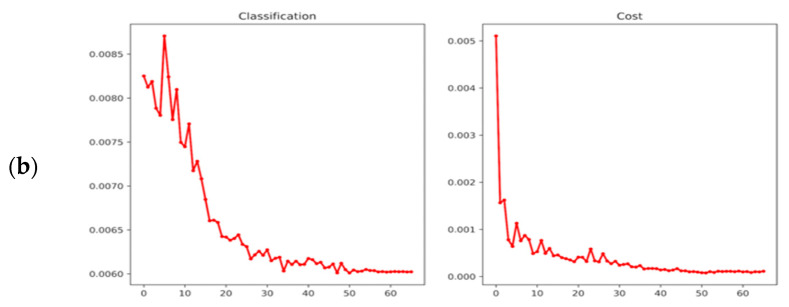
Training curves of (**a**) NEU (**b**) Paint.

Moreover, since the number of images in these two datasets is not large (1800 and 1417, respectively) and the categories are fewer than 10, many epochs are not necessary. We present the training classification loss and cost curves in Figure 8, and we can observe that the models tend to converge under the adopted settings.

### 3.8. Discussions and Comparisons

In this paper, we design a strategy to address the cost-sensitive issue of defect detection by presenting the CS-YOLOv5. Besides the widely adopted YOLO family in manufacturing, another single-stage SSD method [43] is also exploited in the previous literature [44]. Such methods are comparable with YOLO in detection speed, but the settings of the prior box are sensitive and may lead to instability [45]. On the other hand, the two-stage detector Faster-RCNN [4] is also utilized in defect detection [46], which especially perfroms better in denser detection. However, it is slower than single-stage designs due to their higher complexity. More importantly, both of these vanilla versions of the detector cannot process the internal cost-sensitive problem without a specific design, while our CS-YOLOv5 can not only reduce the misclassification cost but also inherits the speed and accuracy advantages.

## 4. Conclusions

To tackle the challenge of cost-sensitive defect detection in manufacturing, in this work, we propose a novel general cost-sensitive learning method called the supervised classification cost-sensitive learning method. Specifically, the misclassification risks represented by a cost matrix are directly integrated into the model optimization. Cost vectors are selected with labels as additional supervised information to train. Based on this approach, we enrich the YOLO family with a cost-sensitive YOLOv5 (CS-YOLOv5). It is a method that reduces the misclassification risk while maintaining the original model structure. We also construct a defect detection dataset from an industrial site. Extensive experiments demonstrate the effectiveness of the proposed CS-YOLOv5 for cost-sensitive defect detection. In the future, we will pay attention to some denser detection tasks which are more sensitive to location information. Hence, future research work can focus on feasible principles and methods for location cost in manufacturing.

## Figures and Tables

**Table 1:** Risk coefficient grouping.

(a): $\lambda_{NP}:\lambda_{PN}:\lambda_{NN}:\lambda_{PP}=200:20:2:1$	(b):$\;\lambda_{NP}:\lambda_{PN}:\lambda_{NN}:\lambda_{PP}=675:45:3:1$
(c):$\;\lambda_{NP}:\lambda_{PN}:\lambda_{NN}:\lambda_{PP}=1600:80:4:1$	(d):$\;\lambda_{NP}:\lambda_{PN}:\lambda_{NN}:\lambda_{PP}=3125:125:5:1$

**Table 2:** mAP and F1-Score with Different Risk Coefficients.

Model	Group	NEU		Paint	
		mAP	F1	mAP	F1
YOLOv5	a	0.76	0.74	0.61	0.63
	b	0.74	0.72	0.61	0.63
	c	0.76	0.74	0.63	0.63
	d	0.74	0.72	0.62	0.63
CS-YOLOv5	a	0.75	0.74	0.63	0.63
	b	0.76	0.74	0.62	0.63
	c	0.76	0.74	0.62	0.62
	d	0.74	0.72	0.65	0.64

**Table 3:** mAP and F1-score with different positive classes.

Model	Group	NEU		Paint	
		mAP	F1	mAP	F1
YOLOv5	1	0.73	0.72	0.63	0.61
	2	0.74	0.72	0.63	0.63
	3	0.74	0.73	0.64	0.65
	4	0.77	0.74	—	—
CS-YOLOv5	1	0.75	0.73	0.61	0.63
	2	0.75	0.72	0.64	0.64
	3	0.78	0.75	0.62	0.61
	4	0.74	0.71	—	—

**Table 4:** mAP and F1-score with different weight ratios.

Weight Ratio	NEU		Paint	
	mAP	F1	mAP	F1
0	0.75	0.73	0.61	0.63
0.25	0.74	0.72	0.61	0.63
0.5	0.72	0.70	0.61	0.64
0.75	0.76	0.73	0.63	0.63
1	0.68	0.67	0.59	0.62
1.25	0.68	0.66	0.60	0.61
1.5	0.68	0.66	0.59	0.59

**Table 5:** The cost performance on NEU with different batch sizes.

Batch size	64	128	230
cost (×10^−3^)	0.48	0.47	0.47

## Data Availability

The datasets used and/or analyzed during the current study are available from the corresponding author on reasonable request.
